# Species-Specific Interactions of *Bacillus* Innocula and Arbuscular Mycorrhizal Fungi Symbiosis with Winter Wheat

**DOI:** 10.3390/microorganisms8111795

**Published:** 2020-11-16

**Authors:** Thomas I. Wilkes, Douglas J. Warner, Veronica Edmonds-Brown, Keith G. Davies

**Affiliations:** 1 Department of Psychology, Sport and Geography, School of Life and Medical Sciences, University of Hertfordshire, College Lane Campus, Hatfield, Hertfordshire AL10 9AB, UK; v.r.edmonds-brown@herts.ac.uk (V.E.-B.); k.davies@herts.ac.uk (K.G.D.); 2 Agriculture and Environment Research Unit, School of Life and Medical Sciences, University of Hertfordshire, College Lane Campus, Hatfield, Hertfordshire AL10 9AB, UK; d.j.warner@herts.ac.uk

**Keywords:** arbuscular mycorrhizal fungi, plant growth promoting rhizobacteria, *Bacillus*, tillage, symbiosis

## Abstract

Arbuscular mycorrhizal (AM) fungi establish close interactions with host plants, an estimated 80% of vascular plant species. The host plant receives additional soil bound nutrients that would otherwise not be available. Other components of the microbiome, such as rhizobacteria, may influence interactions between AM fungi and the host plant. Within a commercial arable crop selected rhizobacteria in combination with AM fungi may benefit crop yields. The precise nature of interactions between rhizobacteria and AM fungi in a symbiotic relationship overall requires greater understanding. The present study aims to assess this relationship by quantifying: (1) AM fungal intracellular root structures (arbuscules) and soil glomalin as an indicator of AM fungal growth; and (2) root length and tiller number as a measure of crop growth, in response to inoculation with one of three species of *Bacillus*: *B. amyloliquefaciences*, *B. pumilis*, or *B. subtilis*. The influence of soil management, conventional (CT) or zero tillage (ZT) was a further variable evaluated. A significant (*p* < 0.0001) species-specific impact on the number of quantifiable AM fungal arbuscules was observed. The inoculation of winter wheat (*Triticum aestivum*) with *B. amyloliquefaciences* had a positive impact on AM fungal symbiosis, as indicated by an average of 3226 arbuscules per centimetre of root tissue. *Bacillus subtilis* increased root length significantly (*p* < 0.01) but decreased fungal symbiosis (*p* < 0.01). The inoculation of field soils altered the concentration of glomalin, an indicator of AM fungal growth, significantly (*p* < 0.00001) for each tillage treatment. The greatest increase was associated with *B. amyloliquefaciences* for both CT (*p* < 0.0001) and ZT (*p* < 0.00001). *Bacillus subtilis* reduced measured glomalin significantly in both tillage treatments (*p* < 0.0001 and *p* < 0.00001 for CT and ZT respectively). The interaction between rhizobacteria and AM fungi is variable, being beneficial or detrimental depending on species. This relationship was evident in both tillage treatments and has important implications for maximizing symbiosis in the crop plant-microbiome present in agricultural systems.

## 1. Introduction

Arable farming relies on supplementary nutrient application to improve and maintain crop development [1]. Arbuscular mycorrhizal (AM) fungi may improve the efficiency of soil nutrient exchange via close symbiotic relationships formed with the host plant. Intracellular root cortical fungal arbuscules allow enhanced assimilation of the soil nutrients obtained by fungal mycelia radiating away from the plant rhizosphere, in exchange for plant derived photosynthetic carbohydrates [2]. An increase in nutrient uptake efficiency potentially decreases dependency on supplementary nutrient inputs. Furthermore, it reduces crop susceptibility to environmental stress such as drought and increased soil salinity [3]. AM fungi also play crucial roles in soil chemistry. Soil carbon sequestration is enhanced through the production and deposition of glomalin, a glycoprotein [4]. The adhesive properties of glomalin coupled with the physical action of mycelia improves the stability of soil microaggregates (<250 µm particle size) [5] resulting in a more stable soil structure less prone to erosion [6]. AM fungal spores are thought to be more resilient to soil disturbance [7] than fungal mycelia, the main source of plant root inoculum [8]. As AM fungi are more abundant in top-soils (<10 cm from the soil surface) [9], soil inversion in conventional tillage (CT) practices break the delicate mycelial networks reducing AM fungi abundance. The abundance of AM fungi may be enhanced by less invasive land management practices such as zero tillage (ZT) [2]. Other components of the soil microbiome, bacteria, have the potential to influence crop growth and AM fungi and crop symbiosis. The nature of this relationship and the impact of crop management variables such as tillage regime, is less well established.

Plant growth promoting rhizobacteria (PGPR) impact plant development and soil fertility [10]. Of particular interest are mycorrhizal helper bacteria (MHB) that stimulate the formation of mycorrhizas and promote the existing plant-fungal symbiotic relationship further [11]. *Azospirillum*, *Pseudomonas*, *Rhodococcus*, and *Bacillus* are genera with known MHB properties. Mycorrhizal helper bacteria have high specificity towards individual species of mycorrhizal fungi, leading to species specific enhancement while other fungal species may simultaneously be inhibited [12]. Mycorrhizal growth promotion, via MHB, promotes increased fungal-plant symbiosis through promoted extensions of mycelia [12] and provides a protective effect when mycorrhizal mycelia proliferate towards root structures and during root colonisation [13]. This protective and improved mycorrhizal growth property of MHB have been studied in field trials in low input agroforestry [11,14]. While mycorrhizal associations in agroforestry are typically ectomycorrhizal, the effect is similar to the branching and pre-symbiotic mechanisms of AM fungi via mycelial growth [13]. Carvalho et al. [15] conclude that MHB are influential in the establishment of mycorrhizal symbiosis with many host plants. Importantly, they are an area that requires further study, a sentiment later echoed by [13].

Mycorrhizal spores may require the presence of MHB to germinate [13,16]. This is thought to be due to the production of the carbohydrate raffinose that the developing spores utilize as a carbon source [17]. Furthermore, some bacterial taxa have been found to live in the cytoplasm of mycorrhiza as endobacteria [18]. Endobacteria are biotrophic towards AM fungi, AM fungi are biotrophic of the host plant and as such, a tripartite relationship has been suggested [18,19]. Early work by Duponnis [20] suggests that the actions of MHB soften the structure of plant cell walls, allowing symbiotic fungi easier access to facilitate the formation of intracellular structures. Cruz and Ishii [21] note that AM fungi have bacteria stored within their spores, AM fungi-associated bacteria (AMB), in order to aid their establishment in plant symbiosis. The relationship that exists with AM fungi and AMB has important implications for agroecosystems and the enhancement of crop growth [18,22]. The impact of crop management and soil tillage on this relationship is relatively unknown. Preliminary work by [16] report on the interactive relationship between AM fungi and AMB in culture dependent and molecular sequencing approaches. They highlight *Sphingobacterium* as being greatest in abundance with associated AM fungi. *Sphingobacterium* are reported to have plant growth promoting capabilities of their own. Furthermore, they demonstrate an inhibitory or destructive effect to many other genera of bacteria, including those that negatively impact AM fungi development [23]. While this is an important step in understanding the associations between AMH and AM fungi, the study by [16] was unable to comment on how different soil management strategies influence the relationship between AMB and AM fungi.

The exact abundance, diversity, and roles of MHB are still unclear and require further investigation. MHB induced mycelial growth increases the probability of mycorrhizal fungi encountering a plant root system which directly correlates to an increase in mycorrhizal–host interactions [11]. However, in vitro studies have not always been able to replicate the MHB and mycelial interactions in the field environment [11,24]. Further study of the relationship between MHB and mycorrhiza in early mycelial growth and development in field extracted soil samples is required. Deveau et al. [25] produced in vitro evidence that early mycelial growth requires thiamine (vitamin B1), typically available under field conditions. *Paenibacillus validus* exists in symbiosis with *Rhizophagus intraradices* providing the AM fungi with thiamine during this critical early growth stage [26]. It is clear from the published literature that a multitrophic community of microbial interactions exists involving the host plant, its AM fungal symbiont, and rhizosphere bacteria. The role of selected AM fungi and selected rhizosphere bacteria on promoting plant growth is well established. The role of MHB and their interaction with AM fungi is however, poorly understood.

The current study investigates the impact of selected rhizobacteria species on AM fungal abundance and host plant development in the presence and absence of tillage by soil inversion. Specifically: (1) the effect of three species of rhizosphere bacteria on winter wheat (*Triticum aestivum*) root colonisation by AM fungi; (2) the relationship between selected rhizosphere bacteria and AM fungi as a time dependent variable; (3) wheat plant growth (quantified via tiller length, tiller numbers, and root length) in relation to AM fungal root colonisation and the formation of root arbuscules; (4) the impact of tillage (conventional, CT or zero, ZT) on selected rhizobacteria species and AM fungi and their relationship with wheat growth. It utilizes a number of attributes including intracellular root cortical arbuscules and soil glomalin as indicators of AM fungal abundance, coupled with plant root length and tiller number, to determine the impact on crop development. The results are discussed in the context of potential value to be derived from the supplementary application of selected rhizobacteria in crop systems to promote AM fungal abundance and crop development.

## 2. Materials and Methods

### 2.1. Samples Sites

The study site consisted of two commercial farms in Hertfordshire, UK: Farm A (near Hitchin, nearest climate station Rothamsted) mean annual rainfall 712.3 mm, mean minimum, and maximum annual temperature 6.0–13.7 °C; Farm B (Hatfield, nearest climate station Rothamsted—same weather parameters as Farm A) [26]. Both were commercially managed farms, with current management undertaken over a minimum period of eight years at the time of sampling. Farm A implemented CT, soil inversion with a moldboard plough to a depth of 20 cm. Farm B practiced ZT by direct seed sowing (John Deere^®^ 750A direct drill, Illinois, USA). The methodology described by Brown and Wherrett (2015) [27] determined that the soil sampled from both farms was sandy loam. Each field in Farm A and B was selected based on the presence of a comparable crop history, and type and application rate of supplementary crop nutrients, with both farms following RB209 [28] recommendations for winter wheat on a sandy loam soil.

### 2.2. Rhizobacteria and AM Fungi Inoculum

Rhizobacteria were sourced from a commercially available mixture (Tribus^®^ Original, Impello Biosciences, Fort Collins, Colorado, USA) consisting of *B. subtilis*, *B. pumilis*, and *B. amyloliquefaciens* at a combined cfu count of 10 billion per millilitre. The three species of Gram-positive *Bacilli* were confirmed in the laboratory by API^®^ (bioMerieux^®^ Ltd, Marcy l’Etoile, ARA, France) colour guides. Each *Bacilli* spp. was extracted from an isolated colony of pure growth samples obtained from serial dilutions and streak plate purification before analysis with API^®^ 50 CH (bioMerieux^®^ Ltd, Marcy l’Etoile, ARA, France) in triplicate. The identity was then confirmed with reference to the API^®^ database (bioMerieux^®^ Ltd, Marcy l’Etoile, ARA, France). The AM fungal inoculum was cultured to a total mass of 5 g in 200 mL nutrient broth for 1 week and homogenised with an electric blender for 1 min. The species identity was confirmed by Eurofins Scientific^®^ (Wolverhampton, West Midlands, UK) as *R. intraradices*.

### 2.3. Impact of Rhizobacteria on AM Fungi Root Arbuscule Number and Winter Wheat Growth

Twenty-four winter wheat plants (four treatments: three *Bacilli spp*. and control; six replicates per treatment) were grown during September 2019 in plastic pots (6 cm radius, 9 cm depth) under glasshouse conditions (20 °C, 37% relative humidity, 15,260 lux) in a 25% J Arthur Bowers^®^ compost (Westland Ltd, Alconbury, Cambridgeshire, UK): 75% J Arthur Bowers^®^ Top Soil w/w mixture with 20 mL of water applied twice weekly. Isolated 10,000 cfu mL^−1^ *Bacilli spp*. rhizobacteria (*B. subtilis*, *B. pumilis*, or *B. amyloliquefaciens*) were applied at a rate of 1 mL to the soil surface of each winter wheat plant replicate every seven days for six weeks in total. The wheat plants (GS21 [29]) were destructively sampled, the root length measured before staining using the protocol developed by Wilkes et al. [30] to obtain AM fungi arbuscule counts. Samples were fixed in a 10% formaldehyde, 50% alcohol, 5% acetic acid *v*/*v* (FAA) solution for 24 h, autoclaved in deionized water and incubated at 60 °C in 5% *v*/*v* hydrochloric acid for 1 h. Roots were divided into 5 × 1 cm sections and stained with 10% *v*/*v* Sheaffer^®^ Blue ink (Providence, RI, USA) in 5% glacial acetic acid for three minutes, subject to root squash and viewed under a light microscope at ×100 magnification. Finally, the number of tillers present in the above ground part of the winter wheat plant was recorded for each replicate. A second set of plants (two tillage treatments, five inoculum treatments (three *Bacilli spp*. AM fungi and control); five samples; three replicates per sample, *n* = 300) were grown for 15 weeks before sampling.

### 2.4. Impact of Rhizobacteria on Soil Glomalin

Glomalin, an indicator of AM fungal growth, was quantified from 25 × 100 g replicates of field sampled soil for the two tillage treatments respectively. Soil (5 kg) was extracted from within 10 cm of the soil surface in the centre of each field post wheat harvest during August 2019. The soil was placed into 500 mL autoclavable plastic beakers, then into trays in groups of five, with 20 mL of water applied per beaker twice weekly while under controlled glasshouse conditions (20 °C, 37% humidity, 15,260 lux). A total volume of 2 mL, 10,000 cfu of each of the three isolated rhizobacteria *Bacilli spp*. (*B. subtilis*, *B. pumilis*, or *B. amyloliquefaciens*) or *R. intraradices* AM fungal inoculum was applied to the soil surface of each respective replicate every seven days for six weeks. Control samples received an application of sterile phosphate buffer solution. Glomalin was extracted from each soil sample via a modified methodology from Wright and Upadhyaya [4] to measure total soil glomalin (TSG). Briefly, 1 g of soil was suspended in 8 mL of 50 mM trisodium citrate dihydrate (Thermo Fisher Scientific^®^, Loughborough, Leicestershire, UK) and maintained at 121 °C/15 psi in an autoclave for 60 min. Soils were then centrifuged at 1000× *g* for 2 min to remove suspended soil particles. The supernatant was further centrifuged at 6800× *g* for 10 min a total of three times to remove impurities within the sample of which 1 mL was then extracted for use in the Bradford Protein Assay (Coomassie Protein Assay Reagent, Thermo Fisher Scientific^®^, Loughborough, Leicestershire, UK) [31] using a Cecil 1021^®^ photo-spectrometer (Cambridge, UK) at an absorbance of 595 nm.

### 2.5. Statistical Analysis

Statistical analyses were conducted using R commander^®^ (Hamilton, ON, Canada). The mean and standard error was calculated for each set of sample data. Paired two-tail *t*-tests of equal variance were employed for post-hoc null hypothesis testing from a single factor ANOVA for differences between *Bacilli* spp. coupled with a Pearson’s Chi squared test. Further paired two-tail *t*-tests of unequal variance were applied to samples between tillage treatments, as were Bonferroni corrections. Statistical significance was determined by *p*-values ≤ 0.05.

## 3. Results

**Figure 1:**
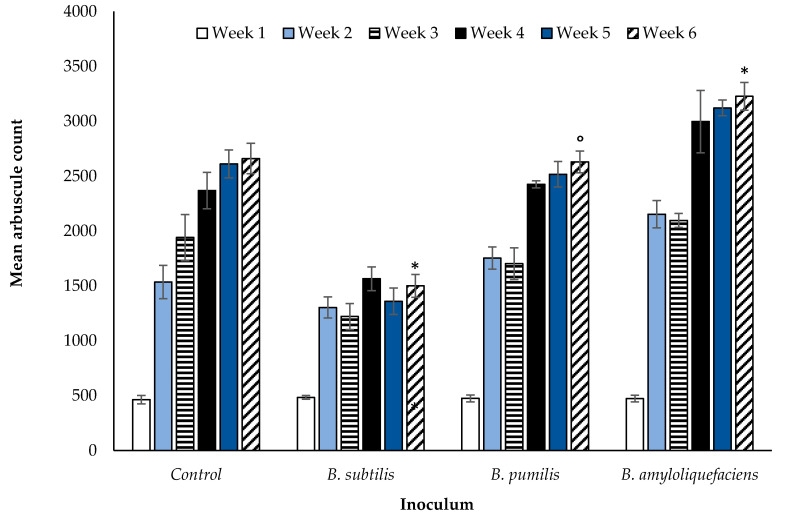
Average (*n* = 270 overall) arbuscule count for inoculated winter wheat over a six week period. Significant (*p* < 0.0001) for single factor ANOVA. TRUE Bonferroni corrections (*), FLASE Bonferroni corrections (°). Error bars = one standard error of the mean (SEM).

**Figure 2:**
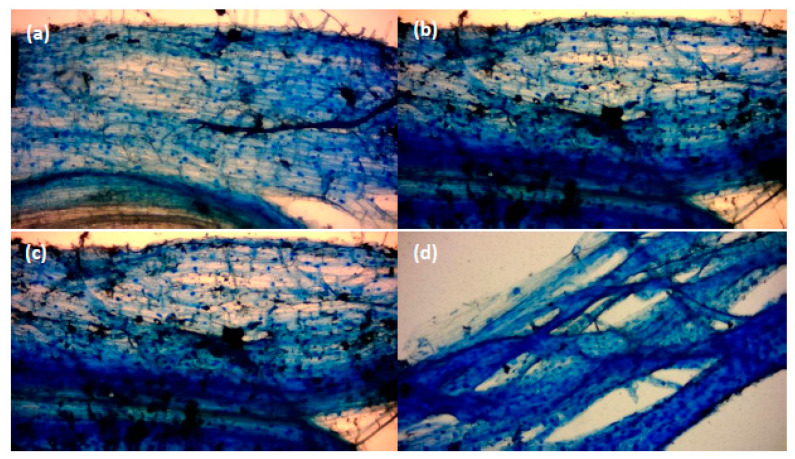
Winter wheat root sections at two weeks post germination stained with Sheaffer^®^ blue: (**a**) control, (**b**) *B. subtilis*, (**c**) *B. pumilis*, and (**d**) *B. amyloliquefaciens*. Image taken with an Apex^®^ microscope (Chippenham, Wiltshire, UK) at a total magnification of ×100 and a Bresser^®^ HD (Rhede, Germany) microscope camera.

The weekly inoculation of winter wheat with one of three selected *Bacillus spp*. rhizobacteria (Section 2.3) had positive (*B. amyloliquefaciens*), negative, (*B. subtilis*), and neutral (*B. pumilis*) effects on the density of AM fungal arbuscules within a 1 cm section of root tissue (Figure 1) (*p* < 0.0001, df: 3.16, F value: 11.55, F critical: 3.24, single factor ANOVA). This is illustrated visually in Figure 2. When applied to developing winter wheat plants, *B. subtilis* reduced the number of AM fungi root arbuscules (*p* < 0.0001, df: 7, t.stat: 6.69, unequal variance *t*-test). Arbuscules were noted to increase in response to the application of *B. amyloliquefaciens* (*p* < 0.01, df: 8, t.stat: −3.02, unequal variance *t*-test). No significant impact was observed in response to the application of *B. pumilis*, indicative of a neutral interaction.

During week one there were no observable difference, irrespective of the bacterial treatment. A post-hoc two-tailed paired *t*-test of equal variance performed post ANOVA indicated significance at week six. The Bonferroni correction factor indicated TRUE corrections for *B. subtilis* and *B. amyloliquefaciens*, indicating these two *Bacilli* spp. influence the number of root arbuscules. A FALSE correction for *B. pumilis* inoculation compared with the control sample suggests the effect of this species on observable AM fungal root arbuscules is neutral.

**Figure 3:**
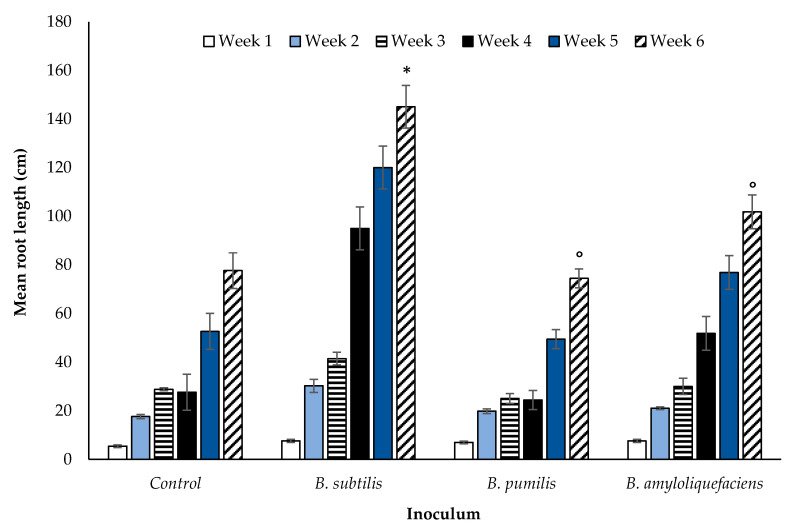
Average (n = 270 overall) root length for inoculated winter wheat over a six week period. Significant (*p* < 0.00001) for single factor ANOVA. TRUE Bonferroni corrections (*), FLASE Bonferroni corrections (°). Error bars = one SEM.

The measured root length of winter wheat grown in compost (Section 2.3) was significantly different between inoculant species and the control (*p* < 0.00001, df: 3.16, F value: 21.73, F critical: 3.29, single factor ANOVA) (Figure 3). The greatest increase in root length resulted from the application of *B. subtilis* (*p* < 0.0001, df: 8, t.stat: −5.87, unequal variance paired *t*-test) (Figure 2). A post-hoc 2 tailed, paired *t-*test of equal variance performed post ANOVA showed significant differences at week six. A Pearson Chi squared analysis between *Bacilli* spp. indicated further that the impact on root length was significantly different (*p* < 0.00001) between species. This effect was neutral for *B. pumilis* and *B. amyloliquefaciens*, as confirmed by a Bonferroni correction of FALSE. An impact was evident for *B. subtilis* (Bonferroni correction of TRUE) in comparison with the control sample.

**Figure 4:**
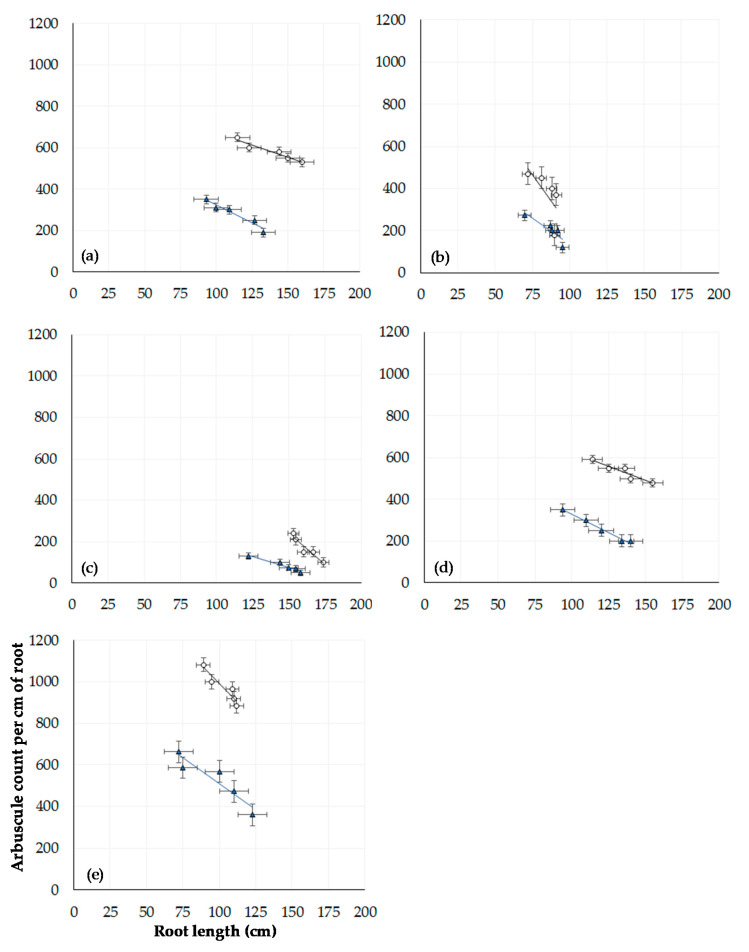
Relationship between root length and arbuscule count (n = 300) at week 15 in 60 winter wheat plants grown in field extracted soil managed under either conventional (blue triangle) or zero tillage (white circle): (**a**) control with zero inoculant, (**b**) AM fungi, (**c**) *B. subtilis*, (**d**) *B. pumilis*, and (**e**) *B. amyloliquefaciens*. Error bars = one SEM.

The data presented in Figure 3 shows the impact on root length of winter wheat plants grown in compost when inoculated with *Bacillus* spp. The relationship between root length and arbuscule number in inoculated wheat plants grown in field extracted soil from either CT or ZT managed fields is shown in Figure 4. A negative relationship between root length and arbuscule number is observed for each treatment, indicating an increase in arbuscular density per unit of root length. Greater numbers of root arbuscules were consistently observed in ZT soils across all inoculants (*p* < 0.00001, df: 7.32, F value: 12.53, F critical: 2.31, single factor ANOVA). Statistical significance was also observed between root length across all inoculations (*p* < 0.0001, df: 7.32, F value: 10.86, F critical: 2.31, single factor ANOVA). Soils inoculated with *B. amyloliquefaciens* had the greatest number of observable arbuscules per 1 cm root section. In contrast, lower numbers of arbuscules were observed in the CT extracted soils for each *Bacillus* spp. (*p* < 0.00001, df: 7.32, F value: 6.45, F critical: 2.31, single factor ANOVA) and per unit increase of root length.

**Figure 5:**
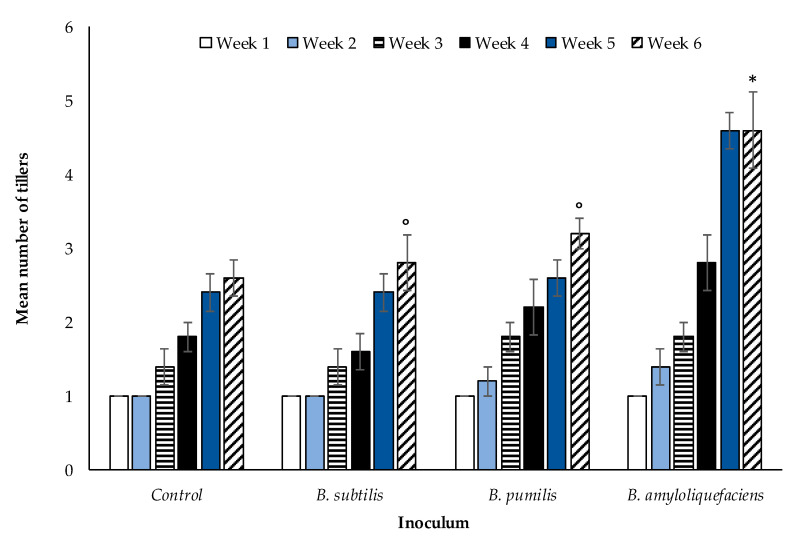
Average (n = 270 overall) number of tillers per plant for inoculated winter wheat over a six week period. Significant (*p* < 0.005) for single factor ANOVA. TRUE Bonferroni corrections (*), FLASE Bonferroni corrections (°). Error bars = one SEM.

In addition to root length, the number of tillers (Section 2.3) differed in response to the three species of rhizobacterial inoculants over a six week period (*p* < 0.005, df: 3.16, F value, 6.51, F critical: 3.24, single factor ANOVA) (Figure 5). Tiller number increased significantly in the *B. amyloliquefaciens* treatment (*p* < 0.006, df: 6, t.stat: −3.54, unequal variance paired *t*-test). The Bonferroni correlation factor was TRUE for *B. amyloliquefaciens*, indicative of this significant effect. *Bacillus subtilis* and *B. pumilis* did not affect the number of tillers significantly relative to the control, further substantiated by a FALSE Bonferroni correlation.

**Figure 6:**
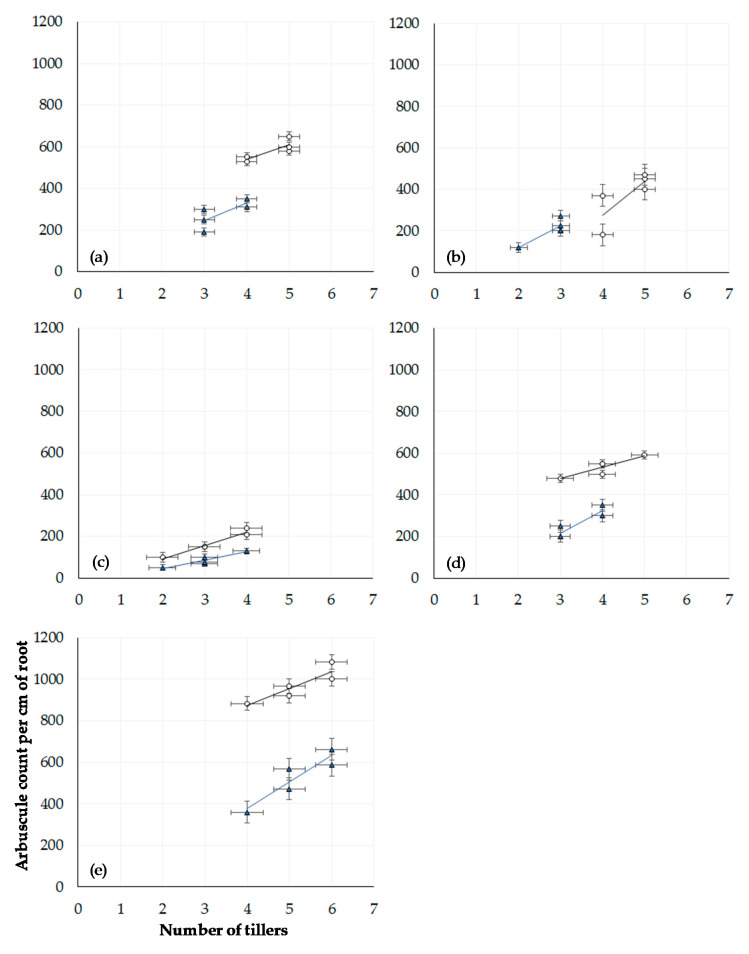
Relationship between tiller number and arbuscule count (n = 300) at week 15 in 60 winter wheat plants grown in field extracted soil managed under either conventional (blue triangle) or zero tillage (white circle): (**a**) control with zero inoculant, (**b**) AM fungi, (**c**) *B. subtilis*, (**d**) *B. pumilis*, and (**e**) *B. amyloliquefaciens*. Error bars = one SEM.

The impact on tiller number of winter wheat plants grown in compost when inoculated with *Bacillus* spp. is shown in Figure 5. The relationship between tiller number and arbuscule number in inoculated wheat plants grown in field extracted soil from either CT or ZT managed fields is given in Figure 6. In contrast to root length, a positive relationship between the number of tillers and counts of arbuscules in root tissue is observed for each treatment, with wheat grown in ZT extracted soils producing a greater quantity of tillers per plant in response to each inoculant (*p* < 0.00001, df: 7.32. F value: 6.57, F critical: 2.31, single factor ANOVA) compared with wheat grown in CT soil (*p* < 0.00001, df: 7.32. F value: 9.38, F critical: 2.31, single factor ANOVA).

**Figure 7:**
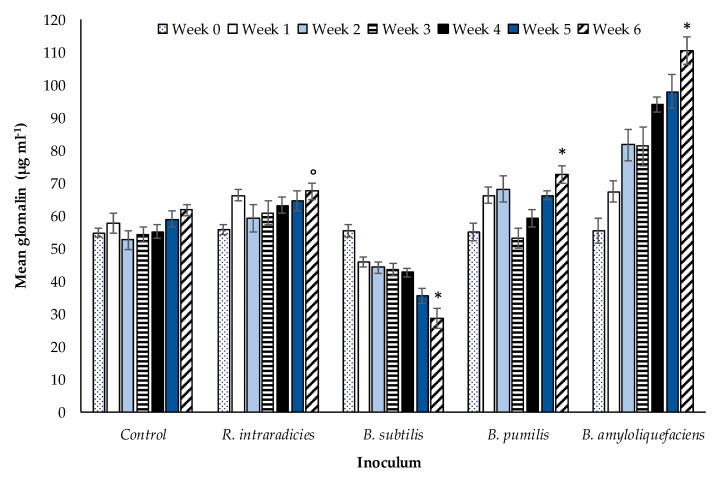
Mean (n = 175 overall) glomalin concentration (µg mL^−1^) comparing inoculated and a non-inoculated control sandy loam top soil (<10 cm) extracted from a ZT managed field over a six week period under controlled glasshouse conditions. TRUE Bonferroni corrections (*), FLASE Bonferroni corrections (°). Error bars = one SEM.

**Figure 8:**
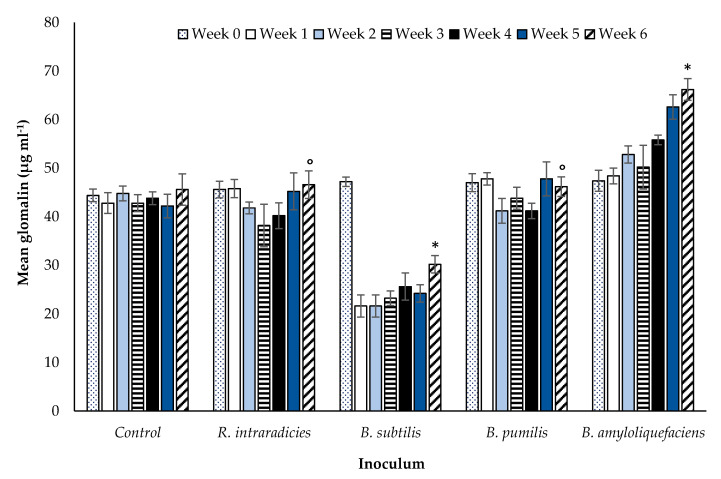
Mean (n = 175 overall) glomalin concentration (µg mL^−1^) comparing inoculated and a non-inoculated control sandy loam top soil (<10 cm) extracted from a CT managed field over a six week period under controlled glasshouse conditions. TRUE Bonferroni corrections (*), FALSE Bonferroni corrections (°). Error bars = one SEM.

The final part of the experiment assessed the impact of tillage on the relationship between AM fungi and the three selected rhizobacterial species using soil glomalin (Section 2.4) as an indicator of AM fungal symbiosis. Soil without vegetation cover was sampled from the ZT field site (Figure 7) and the site practicing CT (Figure 8) and inoculated with selected *Bacillus* spp. and *R. intraradices*. Positive and/or negative variances in quantified glomalin were compared against control samples with no inoculation. After a period of six weeks, soil glomalin differed significantly (*p* < 0.00001, df: 4.30, F value: 15.80, F critical: 2.69, single factor ANOVA) between the three rhizobacterial inoculants in ZT soils (Figure 7).

Inoculation with *B. amyloliquefaciens* increased the concentration of soil glomalin relative to the other treatments, being significantly greater (*p* < 0.00001, df: 8, t.stat: −9.48, unequal variance paired *t-*test) than both the control and the other two *Bacillus* species assessed. *Bacillus pumilis* also increased soil glomalin significantly relative to the control (initial, day zero) treatment suggesting that in addition to *B. amyloliquefaciens*, this species has a positive impact on AM fungal growth. In contrast, soil glomalin decreased significantly (*p* < 0.00001, df: 7, t.stat: 7.29, unequal variance paired *t*-test) relative to the control in the *B. subtilis* treatment. The Bonferroni correction factor indicated TRUE correlations for all *Bacillus spp*., that they significantly affected AM fungal growth. A FALSE correlation was noted for AM fungal inoculation alone in comparison with the control sample, suggesting no significant impact on AM fungal growth. The impact of the three selected rhizobacterial treatments on soil glomalin levels in the CT treatment (Figure 8) followed comparable patterns to the ZT treatment.

Soil glomalin was significantly different (*p* < 0.00001, df: 4.30, F value: 21.26, F critical: 2.69, single factor ANOVA) between the three rhizobacterial inoculants after six weeks. Similarly to the ZT treatment, the greatest increase in glomalin was associated with the inoculation of soil with *B. amyloliquefaciens* (*p* < 0.0001, df: 8, t.stat: −6.04, unequal variance paired *t*-test). Glomalin declined in response to the application of *B. subtilis* (*p* < 0.00001, df: 7, t.stat: 7.29, unequal variance paired *t*-test). No significant difference was observed in soil inoculated with *B. pumilis* or *R. intraradices* relative to the control (*p* = 0.62, df: 8, t.stat: −0.48, paired unequal variance *t*-test). The impact of *B. pumilis* towards AM fungal-root associations appears to be neutral in CT extracted soil, but potentially confers a positive effect in ZT soil. Figure 7 and Figure 8 show how soil glomalin concentration differed between the three rhizobacterial inoculant species when applied to soils extracted from field sites practicing different tillage treatments. A comparison of inoculants individually between tillage treatments at week six i.e., *B. subtilis* in CT compared to *B. subtilis* in ZT (Table 1) identifies statistically significant differences between them.

Tillage exerts an impact on the rhizobacterial species present within the wheat plant microbiome (Table 1). Each of these species exerts a different impact (positive, negative, or neutral) on the development of the plant and potential fungal symbionts. The species-specific nature of this interaction appears crucial and worthy of discussion.

## 4. Discussion

The impact of zero and inversion tillage on the relationship between three species of rhizobacteria and AM fungi has been evaluated using two indicators of AM fungal growth: arbuscule count per centimetre of root tissue and quantity of soil glomalin. A further two indicators, wheat plant root length and tiller number, quantify potential secondary impacts on crop growth. Different responses are observed between AM fungi and each of the three rhizobacterial *Bacilli* spp. A positive relationship exists with *B. amyloliquefaciences*, as indicated by an increase in arbuscule count, glomalin production, and tiller number. The influence of *B. pumilis* appears negligible, while that of *B. subtilis* is suppressive to AM fungal growth as indicated from the reduced quantity of arbuscular root structures. The species-specific nature of the relationship between the selected rhizobacteria and AM fungi was not impacted by tillage regime. These results have strong implications for the supplementary application of rhizobacteria to soils for crop growth enhancement.

The application of *B. amyloliquefaciences* increased soil glomalin concentrations over a six week period in both tillage treatments, CT and ZT. It was significantly higher (*p* < 0.00001) at week six compared with the initial samples in both treatments. Glomalin concentrations were however higher in the ZT treatment (111 µg mL^−1^) relative to soils in which CT (66 µg mL^−1^) was applied. The soil inversion used in CT physically damages the AM fungi mycelial networks, reducing both AM fungal abundance and glomalin production [32,33,34]. Existing literature regarding the association of *B. amyloliquefaciences* and the production of soil glomalin from AM fungi is sparse. Glomalin is an attribute unique to AM fungi [35,36,37,38,39,40,41]. Any increase in glomalin indicates an increase in AM fungal biomass since glomalin functions as a structural support molecule to facilitate the growth and penetration of fungal mycelia within soils [38]. Xie et al. [42] also report an increase in AM fungal symbiosis due to the presence of *B. amyloliquefaciences* using other plant species (*Lotus corniculatus*, *Thymus serpyllum*, and *Trifolium repens*) instead of wheat. While their study did not use soil glomalin as an indicator of AM fungal growth, their findings were comparable with *B. amyloliquefaciences* applications noted to increase AM fungal biomass due to its MHB properties, with some literature indicating AM fungi benefit from the presence of *B. amyloliquefaciences* [42,43,44,45,46]. The significant increase in glomalin in soils from both tillage treatments reported in the current study further supports this conclusion. This study also identifies that the full benefit of *B. amyloliquefaciences* application is realized in combination with soil management conducive to the enhancement of AM fungi. A further indication of the positive effect on AM fungal growth attributed to *B. amyloliquefaciences* is the increase in the density of root arbuscules per centimetre of root tissue.

Arbuscules facilitate the transfer of soil nutrients acquired by AM fungal mycelia to the plant roots [32,33,34,35,36,37,38,39,40,41,42,43,44,45], as well as further enhancing glomalin due to increasing the fungal biomass. The greater proliferation of AM fungi root symbiotic structures increases the plant root surface area, allowing for less plant derived resources required for root growth and development to assimilate the same quantity of nutrients. *Bacillus amyloliquefaciens* enhances AM fungi which in turn enhances plant nutrient uptake efficiency, reducing root biomass while simultaneously increasing tiller number. Additional benefits are realized by the soil, including increased microaggregate stability and carbon sequestration [46]. The direct effect of rhizobacteria on soil aggregate stability is unknown. An increase in plant biomass from the inoculation of soils with select *Bacilli spp*. is also reported by [43]. Application of the Bonferroni factor to the data and *Bacilli spp*. reported in Figure 1 finds that *B. amyloliquefaciences* has the largest positive impact on vegetative plant growth as indicated by a significant increase in tiller number. According to [47] *B. amyloliquefaciens* improves nutrient availability to the plant, through increasing the solubilization of soil phosphates. A further benefit of PGPR is the stimulation of phytohormone production that promote, depending on the hormone stimulated, seed germination, immunity to pathogens, enhanced stress tolerance, or the facilitation of seed production [48]. An increase in the enzyme indoleacetonitrilase required for the production of the phytohormone indole-3-acetonitrile, pivotal in the control of stem and leaf formation, by *B. amyloliquefaciens* is noted by [49]. Furthermore, *B. amyloliquefaciens* is reported by [50] and [51] to suppress plant and fungal pathogenic nematodes and fungal pathogens such as *Fusarium* [52]. The AM fungus, *R. irregularis,* is reported to benefit directly from the presence of *B. amyloliquefaciences* [42,43] acting as MHB facilitating colonisation of the host plant. The beneficial impact to the wheat plant from *B. amyloliquefaciens* inoculation observed here supports the observation of [42], with both an increase in glomalin production and root arbuscules evident. The enhancement of AM fungi colonisation then confers a secondary benefit to the plant by increasing nutrient uptake efficiency. This is coupled with the capacity of *B. amyloliquefaciens* to enhance soil phosphate solubilization meaning that overall, crop growth is less likely to be limited by nutrient deficiency. Indeed, the tiller number was highest in association with inoculation of this PGPR species. Although *B. pumilis* is reported [44,53,54] to enhance plant growth, it was not the conclusion based on the data reported here. No interaction with AM fungi, positive or negative, was apparent. The benefit reported [44,53,54] may be through enhanced phytohormone production but in the absence of stimulated AM fungal growth, it did not in this case produce measurable benefits to the above ground vegetative growth of wheat plants. Furthermore, although several authors report a positive relationship between PGPR and AM fungi [55,56] and its subsequent benefit on plant development, the relationship of *B. subtilis* with AM fungi appeared to be antagonistic.

Soil glomalin, an indicator of AM fungal growth, declined in both ZT and CT immediately after the first application of *B. subtilis* suggesting a rapid inhibition of AM fungal growth. A smaller number of quantifiable root arbuscules was also noted. The quantity of soil glomalin was significantly lower after six weeks in both tillage treatments. The root length of the inoculated winter wheat plants increased (Figure 2), however there was no significant effect on tiller number (Figure 3). Many authors report the impact of PGPR on plant growth only, they do not report on the nature of the interaction between the bacteria and AM fungi directly. With respect to the impact on plant growth alone, increased plant auxin production in response to PGPR [48] would be expected to potentially enhance growth of both roots and leaves. *Bacillus subtilis* only stimulated root production. This may be due to two possible mechanisms. Firstly, the observed suppression of AM fungi reduces nutrient availability, preventing an increase in biomass accumulation and further tiller production. Secondly, the increase in root biomass, although stimulated by enhanced auxin synthesis due to *B. subtilis*, is also a mechanism to compensate for this decline in nutrient availability. While *B. subtilis* may have direct beneficial impacts (auxin production, pathogen suppression), the inhibition of AM fungi growth has indirect negative impacts with respect to plant nutrient uptake efficiency. Rahman et al. [57] note that the simultaneous application of *B. subtilus* and AM fungi to basil (*Ocimum basilicum*) under artificially induced saline stress increased plant height, branch number, fresh and dry weight, percent oil content, and yield. The influence of *B. subtilis* on AM fungal root colonisation is not considered, rendering it difficult to distinguish which species, the bacteria or the fungi, is having the dominant effect on plant growth. In response to the application of *B. subtilis* to marigold (*Tagetes erecta*), Flores et al. [54] report a combination of improvements to cosmetic (flower colour enhancement) and selected plant growth characteristics (increased inflorescence number, flower fresh weight). The promotion of plant growth is also noted by Awasthi et al. [58] for sweet wormwood (*Artemisia annua*), for rose geranium (*Pelargonium graveolens*) by Alam et al. [59] and cucumber (*Cucumis sativus*) by Rabab [60]. The studies suggest a benefit to the plant from enhanced phytohormone production, stimulating further vegetative growth and flowering. Although root length increased in the current experiment, this is not necessarily indicative of a beneficial plant response. Since the decrease in glomalin indicates a decrease in AM fungi, the increase in root length is potentially a mechanism instigated by the plant to compensate for diminished AM fungal symbiosis (as indicated by lower glomalin concentration and a decrease in arbuscule count) and a corresponding decline in soil nutrient assimilation efficiency. The wheat plant allocates greater resources to root development in order to compensate for the loss of nutrients derived from the symbiotic relationship with AM fungi. *Bacillus subtilis* reduces nutrient availability to the wheat plant indirectly through suppression of AM fungi growth. The simultaneous lack of increase in above-ground vegetative growth i.e., tiller number supports this inference. The increase in growth of one particular component of the plant does not, in this case, confer an associated beneficial impact to be attributed to the inoculation of the soil with a rhizobacterium. Requena et al. [61] report enhanced mycorrhizal root colonisation of the legume *Anthyllis cytisoides* when naturally occurring PGPR (*Rhizobium spp.* and *Rhizobacterium*) were applied in combination with the AM fungi *G. intraradices*. The opposite effect was observed when applied with *G. coronatum* instead of *G. intraradices*. Inoculation with *G. coronatum* and *Rhizobium* only however, enhanced mycorrhizal root colonisation. The nature of the impact was related to individual species of AM fungi and species-specific combinations of rhizobacteria. The variability in PGPR behaviour as a function of host plant species and soil environmental conditions is also noted by [61]. Xiao et al. [62] offer a further explanation. The reduction of glomalin and quantifiable arbuscules due to *B. subtilis* may be a result of its plant pathogenic fungal biocontrol properties that extend to selected beneficial AM fungi also.

Naturally occurring PGPR may confer species specific fungi suppressing activity with *B. subtilis* singled out by [63] as a valuable naturally occurring biocontrol agent. The precise mechanism of the interaction between *B. subtilis* and AM fungi is not entirely established. Plants respond to fungal pathogen infection by synthesizing enzymes that degrade the carbohydrates chitin and various glucens within the fungal cell wall [64,65]. The release of ‘Myc factors’ [66,67] and short-chain chitin oligomers [68] by AM fungi enable the fungus to be recognized by the host plant as beneficial organisms rather than pathogenic. The plant does not then elicit the secretion of cell wall degrading enzymes in response. A key question is, does *B. subtilis* distinguish between the AM fungi present and plant pathogenic fungi? The results of this study and others suggest that this is not always the case. According to Xiao et al. [62] the application of *B. subtilis* to maize (*Zea mays*) resulted in a 20% reduction in root colonisation by the AM fungus *G. etunicatum*. *Bacillus subtilis* is able to reduce fungal pathogens either indirectly by producing volatile organic compounds (VOCs) that stimulate an induced systemic resistance (ISR) response in adjacent plants [69], and/or directly via antifungal lipopeptides which act as growth inhibitors [52]. Reference [69] notes differences in the VOCs produced by *B. subtilis* compared with *B. amyloliquefaciens*. If an enhanced or different ISR response in the wheat plant occurs due to differences in the VOC profile between the two *Bacillus* species, the continued recognition by the plant of its associated AM fungi through ‘Myc factors’ would be expected and no effect on AM fungi abundance anticipated. The more likely mechanism is a direct suppressive effect due to the secretion of growth inhibitors by *B. subtilis* but not *B. amyloliquefaciens*. Both *Bacillus* species are reported to inhibit fungal growth [48,52] but this is on a species-specific basis. The type of secondary metabolites synthesized, attributed to conferring anti-fungal properties, differ in profile between the two species. *Bacillus amyloliquefaciens* in general synthesizes a broader range including azalomycin F, bacillomycine, iturin and/or surfactin while in *B. subtilis* this is typically arthrobactin and surfactin [70]. According to [70] there is no single secondary metabolite secreted by *B. subtilis* that is not synthesized by *B. amyloliquefaciens* but there is a difference in quantity and which metabolite dominates in the profile. Arthrobactin for example is produced mainly by *B. subtilis*. The uniqueness of this secondary metabolite profile is however not just species specific but also strain specific [61,70], which may in part explain the conflicting findings of other authors such as [60]. Differentiation of the impact of PGPR fungi suppressing secondary metabolites on AM fungi on a species and strain specific basis will be undertaken as future work.

The total PGPR population is a further key factor [61]. Where the number of PGPR are high in a given volume of soil, the negative impact observed may be due to increased competitiveness with other components of the soil microbiota or due to a direct pathogenic effect [62]. Although the inhibition of fungal pathogens is beneficial to a developing crop, the potential inhibition of spore germination and hyphal development of AM fungi is a negative secondary impact. The decline in mean arbuscule count (Figure 1) lends further support to a fungal inhibitory mechanism due to *B. subtilis*, corroborating [62] and [71]. The method of soil management and the use of tillage does not appear to influence the magnitude of the negative effect of *B. subtilis* on AM fungi and soil glomalin concentration (Figure 7 and Figure 8). In reference to the conclusions of [61] if appropriate numbers of *B. subtilis* were present such that the antagonistic impact reported by [62] becomes aligned with the benefits reported in other studies [54,58,60], opportunities to enhance its biocontrol potential while preserving the benefits of enhanced AM fungi colonisation may exist. Future work will be required to determine optimal numbers.

The species-specific nature of the rhizobacteria—AM fungi interaction has been highlighted by a number of authors [43,72]. In a study of AM fungal community succession in agricultural land, Roy et al. [73] using Illumina sequencing [74] of ribosomal DNA (rDNA) identify species of AM fungi from seven families, mainly *Glomeraceae*, *Diversisporaceae*, and *Claroideoglomeraceae*. They note a change in community composition in response to the time elapsed since the previous cultivation. By this rationale, the species composition of ZT and CT soils may also differ although the response to each of the three rhizobacterial species was similar, suggesting that this was not necessarily the case. Future work would benefit from establishing precisely which species of AM fungi were present, and whether the interaction with the three rhizobacterial species varies with different AM fungi, crop plant species or quantity, species and strain of rhizobacteria present in the plant rhizosphere. If differences in AM fungi community structure and species are identified between tillage management practices, appropriate rhizobacterial species mixtures may be tailored to given combinations of agricultural management and crop plant species.

## 5. Conclusions

Selected rhizobacteria are a valuable component of the rhizosphere however it is important that they are not considered in isolation from other beneficial microorganisms, AM fungi especially. The current study has identified that in the case of winter wheat plants, *B. amyloliquefaciences* enhances AM fungal growth and crop plant development. *Bacillus subtilis*, while conferring biocontrol capabilities that may be of potential value within an agricultural system, was in this case detrimental to AM fungal development and the beneficial plant–root symbiotic relationship. Although neither of these two species of rhizobacteria and its relationship with AM fungi was impacted by the type of tillage regime, the supplementary application of PGPR as biofertilizers require careful consideration on a species and potentially management specific basis. *Bacillus amyloliquefaciences* applied in isolation rather than as a multi-species mixture may offer potential as a commercial crop biofertilizer to enhance crop productivity and preserve naturally occurring AM fungi populations. Furthermore, enhanced nutrient uptake has broader environmental benefits such as increased resource use efficiency and reduced nutrient loss. The gathering of further data through for example, more extensive field trials on a crop type, soil management, PGPR species, and strain specific basis is recommended.

## Figures and Tables

**Table 1:** Summary output of two-tailed, unequal variance paired *t*-tests between tillage treatments and each respective inoculant

Inoculant	*p* Value	Degrees of Freedom	*t* Stat
Control	<0.00001	8	9.84
AM fungus	<0.00001	11	9.60
*B. subtilis*	<0.001	12	3.12
*B. pumilis*	<0.0001	8	6.08
*B. amyloliquefaciens*	<0.001	8	3.85

## References

[1] Olfs H.W., Blankenau K., Brentrup F., Jasper J., Link A., Lammel J. (2005). Soil and plant based fertilizer recommendations in arable farming. J. Plant Nutr. Soil Sci..

[2] Begum N., Qin C., Ahanger M., Raza S., Khan M., Ashraf M., Ahmed N., Zhang L. (2019). Role of Arbuscular Mycorrhizal fungi in Plant Growth Regulation: Implications in Abiotic Stress Tolerance. Front. Plant Sci..

[3] Evelin H., Devi T., Gupta S., Kapoor R. (2019). Mitigation of Salinity Stress in Plants by Arbuscular Mycorrhizal Symbiosis: Current Understanding and New Challenges. Front. Plant Sci..

[4] Wright S.F., Upadhyaya A. (1996). A survey of soils for aggregate stability and glomalin, a glycoprotein produced by hyphae of arbuscular mycorrhizal fungi. Plant Soil.

[5] Liu H., Wang X., Liang C., Ai Z., Wu Z., Xu H., Xue S., Liu G. (2020). Glomlain-related soil protein affects soil aggregation and recovery of soil nutrient following natural revegetation on the Loess Plateau. Geoderma.

[6] Geeta-Vaidya S., Wallander H. (2011). The role of glomalin in soil erosion. Sci. World.

[7] Curaqueo G., Acevedo E., Cornejo P., Seguel A., Rubio R., Borie F. (2010). Tillage effects on soil organic matter, mycorrhizal hyphae and aggregates in a Mediterranean agroecosystem. Rev. Cienc. Suelo Nutr. Veg..

[8] Kabir Z. (2005). Tillage or no-tillage: Impact on mycorrhizae. Can. J. Plant Sci..

[9] Sosa-Hernandez M., Leifheit E., Ingraffia R., Rillig M. (2019). Subsoil Arbuscular Mycorrhizal Fungi for Sustainability and Climate-Smart Agriculture: A Solution Right Under Our Feet?. Front. Microbiol..

[10] Raklami A., Bechtaoui N., Tahiri A., Anil M., Meddich A., Oufdou K. (2019). Use of Rhizobacteria and Mycorrhizae Consortium in the Open Field as a Strategy for Improving Crop Nutrition, Productivity and Soil Fertility. Front. Microbiol..

[11] Labbe J., Weston D., Dunkirk N., Pelletier D., Tuskan G. (2014). Newly identified helper bacteria stimulate ectomycorrhizal formation in *Populus*. Front. Plant Sci..

[12] Frey-Klett P., Garbave J., Tarkka M. (2007). The mycorrhiza helper bacteria revisited. New Phytol..

[13] Rigamonte T., Pylro V., Duarte G. (2010). The role of mycorrhiza helper bacteria in the establishment and action of ectomycorrhizae associations. Braz. J. Microbiol..

[14] Schrey S., Schellhammer D., Ecke M., Hampp R., Tarkka M.T. (2005). Mycorrhiza helper bacterium *Streptomyces* AcH 505 induces differential gene expression in the ectomycorrhizal fungus *Amanita muscaria*. New Phytol..

[15] Carvalho A., Tavares R., Cardoso I., Kuyper T. (2010). Mycorrhizal associations in agroforestry systems. Soil Biology and Agriculture in the Tropics.

[16] Agnolucci M., Avio L., Pepe A., Turrini A., Cristani C., Bonini P., Cirino V., Colosimo F., Ruzzi M., Giovannetti M. (2019). Bacteria associated with a commercial mycorrhizal inoculum: Community composition and multifunctional activity as assessed by illumine sequencing and culture-dependent tools. Front. Plant Sci..

[17] Hildebrandt U., Ouziad F., Marner F., Bothe H. (2006). The bacterium *Paenibacillus validus* stimulates growth of the arbuscular mycorrhizal fungus *Glomus intraradices* up to the formation of fertile spores. FEMS Microbiol. Lett..

[18] Bonfante P., Anca I.A. (2009). Plants, mycorrhizal fungi, and bacteria: A network of interactions. Ann. Rev. Microbiol..

[19] Iffis B., Arnaud M., Hijri M. (2014). Bacteria associated with arbuscular mycorrhizal fungi within roots of plants growing in a soil highly contaminated with aliphatic and aromatic petroleum hydrocarbons. FEMS Microbiol. Lett..

[20] Duponnois R. (1992). Les Bactéries Auxiliaires de la Mycorhization du Douglas (*Pseudotsuga menziesii (Mirb.) Franco*) par Laccaria Laccata Souche. Ph.D. Thesis.

[21] Cruz A., Ishii T. (2012). Arbuscular mycorrhizal fungal spores host bacteria that affect nutrient biodynamics and biocontrol of soil-borne plant pathogens. Open Biol..

[22] Bharadwaj D.P., Lundquist P.O., Persson P., Alström S. (2008). Evidence for specificity of cultivable bacteria associated with arbuscular mycorrhizal fungal spores. FEMS Microbiol. Ecol..

[23] Mohammad B.T., Wright P.C., Bustard M.T. (2006). Bioconversion of isopropanol by a solvent tolerant *Sphingobacterium mizutae* strain. J. Ind. Microbiol. Biotechnol..

[24] Deveau A., Labbe J. (2017). Mycorrhiza helper bacteria. Mol. Mycorrhizal Symbiosis.

[25] Deveau A., Brule C., Palin B., Champmartin D., Rubini P., Garbaye J., Sarniguet A., Frey-Klett P. (2010). Role of fungal trehalose and bacterial thiamine in the improved survival and growth of the ectomycorrhizal fungus *Laccaria bicolor* S238N and the helper bacterium *Pseudomonas fluorescens* BBc6R8. Environ. Microbiol. Rep..

[26] UK Meteorological Office (2018). ‘UK Climate Averages’. https://www.metoffice.gov.uk/research/climate/maps-and-data/uk-climate-averages/gcq89t680.

[27] Brown K., Wherrett A. (2019). Measuring Soil Texture in the Laboratory. http://soilquality.org.au/factsheets/soil-texture-measuring-in-the-lab.

[28] Agriculture and Horticulture Development Board (AHDB) (2018). Nutrient Management Guide (RB209), Section 4 Arable Crops.

[29] Lancashire P.D., Bleiholder H., Langeluddecke P., Stauss R., van den Boom T., Weber E., Witzen-Berger A. (1991). A uniform decimal code for growth stages of crops and weeds. Ann. Appl. Biol..

[30] Wilkes T.I., Warner D.J., Edmonds-Brown V., Davies K.G., Denholm I. (2020). A comparison of methodologies for the staining and quantification of intracellular components of arbuscular mycorrhizal fungi in the root cortex of two varieties of winter wheat. Access Microbiol..

[31] Kruger N.J. (2009). The Bradford method for protein quantitation. The Protein Protocols Handbook.

[32] Jiang X., Alan L., Wright X., Wang F., Liang L. (2011). Tillage-induced changes in fungal and bacterial biomass associated with soil aggregates: A long-term field study in a subtropical rice soil in China. Appl. Soil Ecol..

[33] Brito I., Goss M.J., Carvalho M., Chatagnier O., Tuinen D. (2012). Impact of tillage system on arbuscular mycorrhiza fungal communities in the soil under Mediterranean conditions. Soil Tillage Res..

[34] Wilkes T.I., Warner D.J., Davies K.G., Edmonds-Brown V.R. (2020). Tillage, glyphosate and beneficial Arbuscular Mycorrhizal fungi: Optimizing crop management for plant-fungal symbiosis. Agriculture.

[35] Wright S.F., Frankee-Snyder M., Morton J.B. (1996). Time-course study and partial characterization of a protein on hyphae of arbuscular mycorrhizal fungi during active colonisation of roots. Plant Soil.

[36] Rillig M., Wright S., Eviner V. (2002). The role of arbuscular mycorrhizal fungi and glomalin in soil aggregation: Comparing effects of five plant species. Plant Soil.

[37] Lovelock C.E., Wright S.F., Clark D.A., Ruess R.W. (2002). Soil stocks of glomalin produced by arbuscular mycorrhizal fungi across a tropical rain forest landscape. J. Ecol..

[38] Driver J.D., Holben W.E., Rillig M.C. (2005). Characterisation of glomalin as a hyphal wall component of arbuscular mycorrhizal fungi. Soil Biol. Biochem..

[39] Bendini S., Pellegrino E., Avio L., Pellegrino S., Bazzoffi P., Argese E., Giovannetti M. (2009). Changes in soil aggregation and glomalin-related soil protein content as affected by the arbuscular mycorrhizal fungal species *Glomus mosseae* and *Glomus intraradices*. Soil Biol. Biochem..

[40] Adeleke A. (2010). Effect of Arbuscular Mycorrhizal Fungi and Plant Growth-Promoting Rhizobacteria on Glomalin Production. Master’s Thesis.

[41] Prassad M., Chaudhary M., Ramakrishnan S., Mahawer S. (2018). Glomalin: A miracle protein for soil sustainability. Indian Farmer.

[42] Xie L., Lehvavirta S., Timonen S., Kasurinen J., Niemikapee J., Valkonen J. (2018). Species-specific synergistic effects of two plant growth-promoting microbes on green roof plant biomass and photosynthetic efficiency. PLoS ONE.

[43] Nanjundappa A., Bagyaraj D., Sacena A., Kumar M., Chakdar H. (2019). Interaction between arbuscular mycorrhizal fungi and *Bacillus spp*. in soil enhancing growth of crop plants. Fungal Biol. Biotechnol..

[44] Nadeem S.M., Ahmadm M., Zahir Z.A., Javaid A., Ashraf M. (2014). The role of mycorrhizae and plant growth promoting rhizobacteria (PGPR) in improving crop productivity under stressful environments. Biotechnol. Adv..

[45] Liu X., Feng Z., Zhao Z., Zhu H., Yao Q. (2020). Acidic soil inhibits the functionality of arbuscular mycorrhizal fungi by reducing arbuscule formation in tomato roots. Soil Sci. Plant Nutr..

[46] Acar M., Celik I., Gunal H., Acir N., Barut Z.B., Budak M. (2018). Tillage effects on soil organic carbon, microbial biomass carbon and beta-glucosidase enzyme activity in a typic haploxerert soil. Sci. Pap. Ser. A Agron..

[47] Kim M.J., Radhakrishnan R., Kang S.M., You Y.H., Jeong E.J., Kim J.G., Lee I.J. (2017). Plant growth promoting effect of *Bacillus amyloliquefaciens* H-2-5 on crop plants and influence on physiological changes in soybean under soil salinity. Physiol. Mol. Biol. Plants.

[48] Hassan M.K., McInroy J.A., Kloepper J.W. (2019). The Interactions of Rhizodeposits with Plant Growth-Promoting Rhizobacteria in the Rhizosphere: A Review. Agriculture.

[49] Idris E.E., Iglesias D.J., Talon M., Borriss R. (2007). Tryptophan dependent production of indole3-acetic acid (IAA) affects level of plant growth promotion by *Bacillus amyloliquefaciens* FZB42. Mol. Plant Microbe Interact.

[50] Liu Z., Budiharjo A., Wang P., Shi H., Fang J., Borriss R. (2013). The highly modified microcin peptide plantazolicin is associated with nematicidal activity of *Bacillus amyloliquefaciens* FZB42. Appl. Microbiol. Biotechnol..

[51] Yu Z., Xiong J., Zhou Q., Luo H., Hu S., Xia L. (2015). The diverse nematicidal properties and biocontrol efficacy of *Bacillus thuringiensis* Cry6A against the root-knot nematode *Meloidogyne hapla*. J. Invertebr. Pathol..

[52] Khan N., Martínez-Hidalgo P., Ice T.A., Maymon M., Humm E.A., Nejat N., Hirsch A.M. (2018). Antifungal activity of Bacillus species against Fusarium and analysis of the potential mechanisms used in biocontrol. Front. Microbiol..

[53] Chakraborty U., Chakraborty B., Allay S., De U., Chakraborty A. (2011). Dual application of *Bacillus pumilus* and *Glomus mosseae* for improvement of health status of mandarin plants. Acta Horticult..

[54] Flores A., Luna A., Portugal O. (2007). Yield and quality enhancement of marigold flowers by inoculation with *Bacillus subtilis* and *Glomus fasciculatum*. J. Sustain. Agric..

[55] Medina A., Probanza A., Manero F., Azcon R. (2003). Interactions of arbuscular-mycorrhizal fungi and *Bacillus* strains and their effects on plant growth, microbial rhizosphere activity (thymidine and leucine incorporation) and fungal biomass (ergosterol and chitin). Appl. Soil Ecol..

[56] Hernandez J., de-Bashan L., Rodriguez D., Rodriguez Y., Bashan Y. (2009). Growth promotion of freshwater microalga *Chlorella vulgaris* by the nitrogen-fixing, plant growth-promoting bacterium *Bacillus pumilus* from arid zone soils. Eur. J. Soil Biol..

[57] Rahman A.S.S., Abdel-Kader A.A., Khalil S.E. (2010). Response of three sweet basil cultivars to inoculation with Bacillus subtilis and arbuscular mycorrhizal fungi under salt stress conditions. Nat. Sci..

[58] Awasthi A., Bharti N., Nair P., Singh R., Shukla A., Gupta M., Darokar M., Kalra A. (2011). Synergistic effect of *Glomus mosseae* and nitrogen fixing *Bacillus subtilis* strain Daz26 on artemisin content in *Artemisia annua* L.. Appl. Soil Ecol..

[59] Alam M., Khaliq A., Sattar A., Shukla R., Anwar M., Seema S. (2011). Synergistic effect of arbuscular mycorrhizal fungi and *Bacillus subtilis* on the biomass and essential oil yield of rose-scented geranium (*Pelargonium graveolens*). Arch. Agron. Soil Sci..

[60] Rabab M. (2014). Interaction of *Bacillus subtilis* and *Trichoderma harzianum* with mycorrhiza on growth and yield of cucumber (*Cucumis sativus* L.). Int. J. Curr. Res..

[61] Requena N., Jimenez I., Toro M., Barea J. (1997). Interactions between plant-growth-promoting-rhizobacteria (PGPR), arbuscular mycorrhizal fungi and *Rhizobium* spp. in the rhizosphere of *Anthyllis cytisoides*, a model legume for revegetation in Mediterranean semi-arid ecosystems. New Phytol..

[62] Xiao X., Chen H., Chen H., Wang J., Ren C., Wu L. (2007). Impact of *Bacillus subtilis* JA, a biocontrol strain of fungal plant pathogens, on arbuscular mycorrhiza formation in *Zea mays*. World J. Microbiol. Biotechnol..

[63] Schönbichler A., Díaz-Moreno S.M., Srivastava V., McKee L.S. (2020). Exploring the Potential for Fungal Antagonism and Cell Wall Attack by *Bacillus subtilis natto*. Front. Microbiol..

[64] Olanrewaju O.S., Glick B.R., Babalola O.O. (2017). Mechanisms of action of plant growth promoting bacteria. World J. Microbiol. Biotechnol..

[65] Balestrini R., Bonfante P. (2014). Cell wall remodeling in mycorrhizal symbiosis: A way towards biotrophism. Front. Plant Sci..

[66] Catoira R., Galera C., de Billy F., Penmetsa R.V., Journet E.P., Maillet F. (2000). Four genes of *Medicago truncatula* controlling components of a nod factor transduction pathway. Plant Cell.

[67] Parniske M. (2008). Arbuscular mycorrhiza: The mother of plant root endosymbioses. Nat. Rev. Microbiol..

[68] Genre A., Chabaud M., Balzergue C., Puech-Pagès V., Novero M., Rey T. (2013). Short-chain chitin oligomers from arbuscular mycorrhizal fungi trigger nuclear Ca^2+^ spiking in *Medicago truncatula* roots and their production is enhanced by strigolactone. New Phytol..

[69] Farag M.A., Ryu C.M., Sumner L.W., Paré P.W. (2006). GC–MS SPME profiling of rhizobacterial volatiles reveals prospective inducers of growth promotion and induced systemic resistance in plants. Phytochemistry.

[70] Wulff E.G., Mguni C.M., Mansfeld-Giese K., Fels J., Lübeck M., Hockenhull J. (2002). Biochemical and molecular characterization of *Bacillus amyloliquefaciens, B. subtilis* and *B. pumilus* isolates with distinct antagonistic potential against *Xanthomonas campestris pv. campestris*. Plant Pathol..

[71] Desmyttere H., Deweer C., Muchembled J., Sahmer K., Jacquin J., Coutte F., Jacques P. (2019). Antifungal activities of *Bacillus subtilis* lipopeptides to two *Venturia inaequalis* strains possessing different tebuconazole sensitivity. Front. Microbiol..

[72] Thirkell T., Charters M., Elliott A., Sait S., Field K. (2017). Are mycorrhizal fungi our sustainable saviours? Considerations for achieving food security. J. Ecol..

[73] Roy J., Reichel R., Bruggemann N., Hempel S., Rillig M. (2017). Succession of arbuscular mycorrhizal fungi along a 52-year agricultural recultivation’. FEMS Microbiol. Ecol..

[74] Canard B., Sarfati R.S. (1994). DNA polymerase fluorescent substrates with reversible 3′-tags. Gene.

